# From L. C. Ingersoll

**Published:** 1882-10

**Authors:** L. C. Ingersoll


THE SAFEST ANÆSTHETIC KNOWN.—Dr. Richardson says, that methyline bichloride, ten fluid drachms, and absolute methylic alcohol, six fluid drahchms, constitute the safest known anæsthetic when the methylic alcohol is absolutely pure.—*Lancet.*

EDITOR REGISTER.

*Dear Sir*—In your September number of the REGISTER you have noticed the calcarious deposit which I have named *Sanguinary Calculus*, and proposed a new name, and ask, how I like it.

It is best for the progress of dental science that every additional thought and discovery should be carefully examined and placed in its proper category. This I attempted to do in giving name to the dark granular deposit found at the apex, or deep under the gums on the roots of teeth. I think if you had made a little more careful examination of the conditions under which it is found deposited, you would not have written the first line of your criticism, thus: "The roots of teeth involved in abscess often have upon them calcarious deposits." And farther on,—"The calcarious material is, previous to its deposition, held in solution by the fluid pus or sanies that surrounds the root in an abscess."

The fact is, according to my observation, that this peculiar form of deposit is *never found in connection with abscess*, but always in connection with alveolar ulceration—in many cases where there has never been any abscess. The last named cases might be ranked under the head of the so-called Riggs disease. There is a form of alveolar ulceration which is confounded with alveolar abscess; it differs from the Riggs disease in that it originates at the apex of the root instead of the margin of the gum. It differs from abscess in all the characteristics that mark an abscess—causes no swelling, has no circumscribed pocket to hold the pus, and no fistulous canal reaching out from the cavity of confined pus to the surface of the gum. The pus discharged is also different from that discharged from an abscess. It is thin and watery, and almost free from odor. It is from this watery fluid, composed chiefly of *liquor sanguinis*, that the calcarious matter in question is deposited; not from the thick icherous pus of alveolar abscess. The *liquor sanguinis* passing out from the blood through the vascular coatings by exosmosis, bathes freely the surface of the root denuded of its investing membrane, and parts with its lime salts held in solution, because of the greater affinity for the lime composing the tooth structure; and for this reason the root of the tooth becomes the nucleus for the deposit. Coming, therefore, so directly from this normal fluid, the liquor sanguinis, it seemed eminently fitting to me to call the deposit *sanguinary calculous.* The term is thus in harmony with the nomenclature of science, which has given names to other calcarious deposits and concretions, all of which are derived from normal fluids of the body. Thus understood, Mr. Editor, the term does not, as you have asserted, "do violence to truth and science," but harmonizes with both. L. C. INGERSOLL.

